# Multiple Myeloma: A Personal Account of My Journey With the Disease and Response to Teclistamab

**DOI:** 10.7759/cureus.59781

**Published:** 2024-05-07

**Authors:** Mohammad Ahmad, Musa F Zahrani, Ghazi S Alotaibi, Fatimah Alshalati, Ammarah Afzal, Ibrahim N Alrumaih, Rabia Riaz, Muhammad Tayyab

**Affiliations:** 1 Department of Medical Surgical Nursing, College of Nursing, King Saud University, Riyadh, SAU; 2 Department of Medicine, King Khalid University Hospital, King Saud University, Riyadh, SAU

**Keywords:** kappa-free light chain (flc), serum creatinine phosphokinase, b-cell maturation antigen (bcma), relapse/refractory, diagnosis of multiple myeloma

## Abstract

Multiple myeloma (MM) remains an incurable hematologic cancer leading to damage to the bone marrow that causes destructive bone lesions in addition to many other effects. I am a patient with MM who has undergone treatment to date since the diagnosis of this disease in December 2019. This paper reviews the treatments and observations made throughout this period. The salient results of such treatments are discussed in chronological order. During this period, my MM relapsed and then I was introduced to teclistamab treatment. The outcome of teclistamab treatment is quite promising, and I anticipate a longer life at a maintenance dose of this drug with a better quality of life. When writing this article, I am still receiving the teclistamab treatment cycles that maintain a constant normal level of my kappa-free light chain (FLC) and kappa/lambda ratio, with no significant side effects.

## Introduction

Prelude

I, Mohammad Ahmad, am a patient with multiple myeloma (MM), and all the other authors in this article are my physicians who were deeply involved in my treatment from the day of diagnosis till date. I am reviewing my treatments from the day of diagnosis up to the present day and wish to share some significant personal observations on MM.

MM is a treatable but incurable hematologic malignancy that is caused by the outgrowth of monoclonal plasma cells leading to damage in the bone marrow [1]. Plasma cells normally develop from B cells that the body's immune system uses to fight infection and diseases by changing into plasma cells; they are responsible for creating antibodies to help fight diseases. These plasma cells are found mainly in the bone marrow. When they begin to grow in an uncontrolled manner, they start producing abnormal proteins causing destructive bone lesions, kidney injury, anemia, hypercalcemia, and plasmacytomas, which result in tumor growth of plasma cells [2].

Looking at the chronology of the treatment of patients with MM, it is found that a significant advancement was observed since the introduction of immunomodulatory agents (IMiD) in the 1990s, proteasome inhibitors in the 2000s, monoclonal antibodies in the 2010s, and treatments with t cells engineered with chimeric antigen receptor (CAR-T) in the 2020s [3]. Although the majority of MM patients are currently not cured, recent studies encourage longer follow-ups with the result of a disease-free state for a longer period [4]. Following the recommended International Myeloma Working Group (IMWG) criteria for the diagnosis of MM, it requires the presence of one or more myeloma-defining events (MDE) in addition to evidence of either 10% or more clonal plasma cells on bone marrow examination or a biopsy-proven plasmacytoma. MDE consists of established CRAB (hypercalcemia, failure, anemia, or lytic renal bone lesions) features as well as three specific biomarkers: clonal bone marrow plasma cells ≥60%, serum free light chain (FLC) ratio ≥100 (provided involved FLC level is ≥100 mg/L), and more than one focal lesion on MRI [5]. Furthermore, it has been advised that when MM is suspected clinically, patients should be tested for the presence of M proteins using a combination of tests that should include a serum protein electrophoresis (SPEP), serum immunofixation (SIFE), and serum FLC assay [5]. Other important criteria that may be useful in the diagnosis of MM have also been reviewed [6]. Developments in the treatment of newly diagnosed MM have improved the survival age of such patients significantly in the last decade [7]. The initial impact came from the introduction of thalidomide, bortezomib, and lenalidomide [8,9]. Combinations of many other drugs also, like, carfilzomib, pomalidomide, ixazomib, elotuzumab, daratumumab, isatuximab, selinexor, belantamab mafodotin, and CAR-T cell therapies have been developed recently and approved by the Food and Drug Administration (FDA) for the treatment of relapsed MM, promising for an improved outcome [6].

However, standard first-line (induction) therapy for all patients with MM consists of a combination of an injectable proteasome inhibitor (i.e., bortezomib), an oral IMiD (i.e., lenalidomide) and dexamethasone [2]. Thus, my treatment for MM started with the CyBorD (cyclophosphamide, bortezomib, and dexamethasone) cycle treatment (Table 2) and the details of the initial eight cycles of CyBorD are given in Table 3. The FDA recently approved teclistamab, a bispecific antibody that redirects t cells, in patients with relapsed/refractory (R/R) MM who have failed prior lines of standard therapy. Teclistamab, a bispecific antibody, targets both CD3 expressed on t cells and b cells maturation antigen (BCMA) expressed on the surface of myeloma cells [10]. It is the first bispecific antibody directed to T cells to be approved for R/R MM [11,12].

Given the above information, I will now review the status of my MM disease from the day of admission to the hospital for diagnosis up to the present date while writing this manuscript. This journey of more than four years describes the relevant sequential workups, follow-ups, and treatment courses followed throughout. Although much information is available in the literature on the behavior of MM during its course; the present article describes my personal experience with MM that will provide some important information on the course of MM treatment. Available literature suggests that teclistamab can cause some unwanted side effects like cytokine release syndrome (CRS), neurologic toxicity, immune effector cell-associated neurotoxicity syndrome (ICANS), and other severe side effects [13]. However, in my case, it appears that teclistamab has shown a good response as reported in the earlier R/R MM studies [11,12] with no side effects. I hope to have a longer life on a maintenance dose of this drug with a better quality of life.

## Case presentation

I am a 69-year-old Indian male, working as a professor in the College of Nursing, King Saud University, Riyadh, Saudi Arabia. Since mid-2019, I started developing lower back pain that worsened day by day, limiting my daily activities. I developed gradual bilateral pain in the shoulders and ribs as well, especially in the right lower ribs, limiting my movements. Even analgesics were ineffective in relieving pain, and I started sleeping on my left side to minimize pain. Furthermore, I started to lose my appetite, with subjective fever all day, fatigue, and malaise. I lost more than eight kilograms of body weight in three months and had polyuria and nocturia. My thighs started paining while trying to stand up from a sitting posture. I sought medical advice and was seen by neurosurgery and orthopedic clinics. They recommended magnetic resonance imaging (MRI) done on November 11, 2019, and prescribed analgesics that had no relieving effect. Finally, I visited the emergency department of King Saud University Medical City. Based on the above symptoms, I was admitted to the hospital on December 12, 2019, under the internal medicine service as a suspected case of MM for further investigation and diagnosis.

Diagnosis procedures, course of treatment, and salient responses

The date of my MM diagnosis was December 15, 2019, at the age of 64 years. All the laboratory investigations and imaging required for the diagnosis of suspected MM were carried out as summarized in Table 1.

**Table 1:** Workouts at the time of diagnosis for my multiple myeloma Diagnosis workouts consisted of various laboratory tests including serology, immunology, immunohistochemistry, MRI Imaging, and bone marrow biopsy. Normocytic normochromic Anemia was noticed, consistent with involvement by plasma cell myeloma with kappa light chain restriction. Bone biopsy showed few fragments of viable and non-viable bone cells with bone marrow showing scattered interstitial plasma cells. My disease MM was diagnosed on December 15, 2019, at the age of 64 years. Mobilization improved and pain due to spine surgery, almost subsided by the time I was discharged on January 5, 2020. Treatment of MM commenced immediately after the diagnosis on December 15, 2019.

Laboratory tests	Results	Reference values
Urine Bence Jones protein	Positive	---
HIV and hepatitis serology	Negative	---
Hemoglobin	98 g/L	140-170 g/L
Platelet	181.0x10^9^/L	150-350 x 10^9^/L
WBCs	8.600x10^9^/L	4.5-11 x 10^9^ cells/L
Absolute lymphocyte count	0.3x10^9^/L	0.77-4.5 x 10^9^ cells/L
Absolute neutrophil count	7.9x10^9^/L	0-1.2 x 10^9^ cells/L
Creatinine	414 mcmol/L	60-110 mcmol/L
Corrected calcium	3.03 mmol/L	2.2-2.6 mmol/L
Lactate dehydrogenase	185 unit/L	60-160 U/L
Albumin	33.92 gm/L	60-78 g/L
Beta 2 microglobulin	8.44 mg/L	0.7-1.8 mg/L
HIV and Hepatitis	Negative	---
kappa free light chain	18,200.00 mg/L	3.3-19.4 mg/L
lambda	57.10 mg/L	5.71-26.3 mg/L
kappa/lambda ratio	318.74	0.26-1.65
IgA	1.510 gm/L	0.7-3.0 g/L
IgG	11.2 gm/L	6.4-14.3 g/L
IgM	0.212 gm/L	0.2-1.4 g/L
CD38 and CD138	Highlighted multiple small aggregates and diffused clusters of plasma cells with monoclonal kappa restriction were noticed.	
CD56	Positive	---
CD20, CD117, and Lambda	Negative	---
MRI done on November 16, 2019	The diffused abnormal signal intensity of the lumbosacral spine involving vertebral bodies and posterior elements with L3 compression fracture was noticed.	
Spine surgery was done on November 29, 2019	Underwent percutaneous instrumented fusion of L2-L4 and was kept under physiotherapy and dressing.	
Skeletal survey	Multiple, well-defined lytic lesions (punched-out lesions) of various sizes were also observed, scattered throughout the skull. Degenerative changes were noted at the cervical spine with multi-level disc space narrowing at C5-C6 and C6-C7 levels.	
Bone marrow biopsy was done on December 15, 2019	Aspirate showed plasma cells at 4%, and cellularity at 50%; diffused sheets of plasma cell infiltration were noted, mostly of mature plasma cells in addition to interstitial involvement.	
Overall mobilization	was improved and pains almost subsided by the time I was discharged on January 5, 2020.	

All corresponding results of the tests (Table 1) supported the diagnosis of MM as reviewed earlier [5,6]. Furthermore, the hospital treatment course as an inpatient after the diagnosis is briefly summarized in Table 2 along with the plan that was undertaken after discharge from the hospital on January 5, 2020.

**Table 2:** Hospital treatment course as an inpatient after admission and diagnosis of multiple myeloma Discharged from the hospital on January 5, 2020, with a further treatment plan. 1) Was advised to remain on good fluid hydration and intake. 2) To continue on Sodium bicarbonate (2 tabs TID) as per recommendations of the Nephrology clinic. 3) Was encouraged for mobility. 4) To control the pain with Tylenol TID 2 tabs. 5) To follow the spine orthopedic clinic with plaster wound dressing. 6) To continue CyBorD cycle number 2 in the medical oncology day care (MODC) clinic with phlebotomy lab tests beforehand. 7) To take aspirin daily.

Date	Drug/treatment	Administering details
December 13, 2019	Dexamethasone	40 mg po daily x4 days
	Bortezomib	1.5 mg/m^2^ subcutaneous
December 17, 2019	Dexamethasone	1.3 mg/m^2^ po. Thereafter, received 40 mg daily for four days to decrease the load of myeloma.
	Pamidronate	30 mg iv
	Calcitonin	4 units/kg BID for three days with aggressive hydration
	Calcium gluconate	Started intra venous
	Calcium bicarbonate	Started orally
	CyBorD (cyclophosphamide, bortezomib, and dexamethasone)	One cycle was completed twice weekly as an inpatient
	Valacyclovir	500 mg oral BID
	Heparin	Subcutaneous BID

By date July 22, 2020, after discharge, my treatment completed eight CyBorD cycles (Table 3).

**Table 3:** Protocol of the eight cycles of CyBorD (cyclophosphamide, bortezomib, and dexamethasone) treatment received from January 2020 to July 2020 CyBorD cycles consisted of eight cycles (once weekly) with a cycle length of 28 days. ESR: erythrocyte sedimentation rate

Drugs with dose	Days of treatment	Observations
Bortezomib 1.5 mg/m^2^	On days 1, 8, 15, and 22 of the cycle	ESR: 60, 40, 60, and 58, respectively; kappa free light chain (FLC): 18K, 3K, 6K, 7K, 8K, 7K; Calcium: 2.5 mmol/L; Hb: 9.2 g/L; Creatinine: 26 mcmol/L; Platelets: 75.0 x10^9^/L Much improvement was observed in the overall condition. Since the platelets count came down to 75, cyclophosphamide was omitted from day 22 of cycle no. 8. Mobility was good. Future appointments were to be continued as per protocol. Since the FLC value increased from 3K to 7K, carfilzomib was initiated from further cycles.
Cyclophosphamide 300 mg/m^2^	On days 1, 8, 15, and 22 of the cycle	
Dexamethasone 40 mg	Cycle 1 and 2 on days 1-4, 9-12, 17-20; cycle 3 onwards on days 1, 8, 15, 22	
Adalat 30 mg PO BID	Daily	
Valacyclovir 500 mg PO BID	Daily	
Metmorfin 850 mg BID	Daily	
Asprin 81 mg OD	Daily	

**Figure 1:**
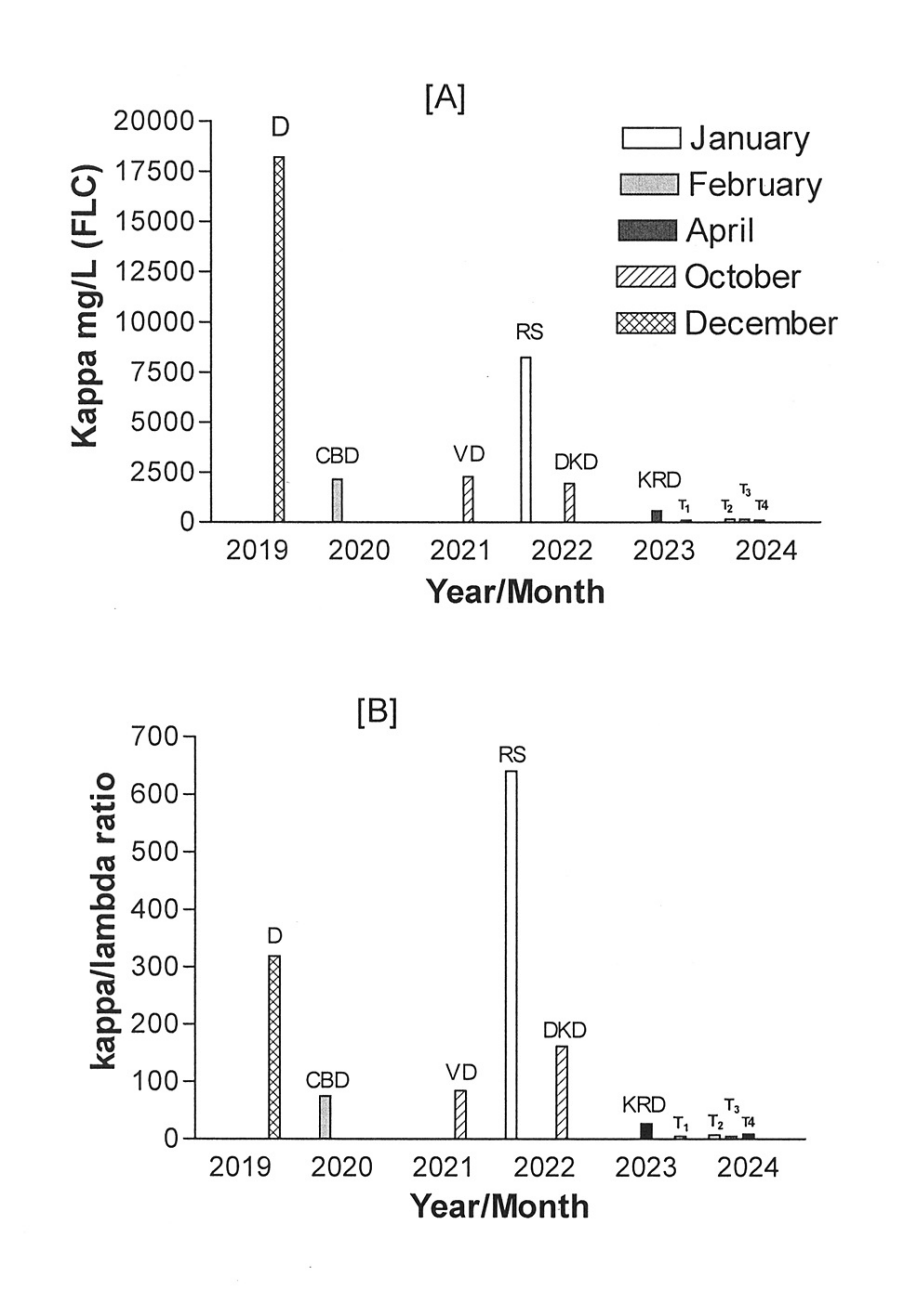
(A) Levels of kappa free light chains (FLC); (B) kappa/lambda ratio from December 2019 to February 2024 during the treatment of multiple myeloma through various protocols of chemotherapy D: the day of diagnosis, December 2019; CBD: CyBorD (cyclophosphamide, bortezomib, dexamethasone) 12 cycles till February 2020; VD: valcade/dexamethasone 12 cycles till October 2021; RS: relapse stage of multiple myeloma due to drug holiday till January 2022; DKD: carfilzomib, dexamethasone, daratumumab nine cycles till October 2022; KRD: carfilzomib-lenalidomide-dexamethasone five cycles till April 2023; T1: teclistamab since May 2023 x8 cycles till December 2023; T2: teclistamab January 2024; T3: teclistamab February 2024; T4: teclistamab March 2024

My kappa FLC and kappa/lambda ratio came down very significantly (Table 3, Figure 1). Thus, from July 23, 2020, to October 21, 2021, I was treated with 12 cycles of bortezomib and dexamethasone and the kappa FLC level was at 2,270 reflecting a stable condition of my MM at that stage (Table 3, Figure 1). Thereafter, I went on a drug vacation from October 22, 2021, to January 22, 2022, after discussing the pros and cons with my treating physician. This resulted in a relapsed/refractory (R/R) MM with a level of kappa FLC increasing to 8,250 (Table 3, Figure 1).

Under such conditions, I was treated with nine cycles of the DKD regimen (carfilzomib, dexamethasone, daratumumab) and this brought my kappa FLC level to 1,940 which was a significant improvement (Table 3, Figure 1). Subsequently, I was switched to treatment with five cycles of the KRD (carfilzomib-lenalidomide-dexamethasone) regimen because of the cycle restriction, and this resulted in a further decrease in the FLC level of kappa to 567 (Table 3, Figure 1).

Thereafter, the kappa FLC level remained at a plateau and did not decrease further. Thus, in May 2023, it was decided to start me on teclistamab. I had a good discussion about teclistamab with my physicians and was well-informed in detail about the mode of action of this drug and also the possible side effects that I could be facing. Subsequently, I was admitted for teclistamab step-up dose therapy to monitor for CRS/ICANS. On day 1, the first dose of 0.06 mg/kg of teclistamab was administered sc. On day 3, 48 hours after the first dose, the second dose of 0.3 mg/kg was also administered sc. The third dose of 1.5 mg/kg teclistamab was administered sc, 48 hours after the second dose. This was the full dose and was administered every week at the Medical Oncology Day Care (MODC) clinic. No side effects were noticed. Before 1-3 hours of all step-up therapies, as well as weekly therapies with teclistamab, a pretreatment was carried out with oral or intravenous administrations of dexamethasone 15 mg; diphenhydramine 50 mg; and acetaminophen 650 mg. After starting teclistamab treatment, no side effects were observed; however, initially, the FLC of kappa did not decrease below the 567 level. But with a delayed response, from August 2023, the kappa FLC level started to fall from 567 to the level of 352 (Figure 1). In December 2023, it came down remarkably to the 1.94 level. It is important to mention here that along with teclistamab therapy, I am receiving monthly IVIG support to have control over getting infections [14]. To date, I am receiving teclistamab (1.5 mg) sc every second week instead of weekly as at the beginning of the teclistamab treatment. This biweekly dosing of teclistamab has been very recently approved by the US FDA [15]. I have no side effects or pain in my ribs or other parts of my body. However, stiffness and frequent mild back pain are probably due to the percutaneous instrumented fusion of L2-L4 spine surgery. To overcome this pain, I take paracetamol as and when required and it gives me relief. I have good mobility now and experiencing a better quality of life.

However, as a prophylactic precaution against HSV/VZV, fungal and PCP infections, I take daily orally the prescribed medicines pantaprazole 40 mg; fluconazole 100 mg; linagliptin 5 mg; acyclovir 400 mg; aspirin 81 mg; multivitamin (renal tab); calcium carbonate 600 mg as calcium; and every alternate day, sulfamethoxazole+trimethoprim 800/160 mg. To date, at the time of writing this article for publication, my condition is stable, with no side effects, and my age has now crossed 69 years.

Results

There are many laboratory data and results related to the prognosis and treatment of my MM, and it is impossible to include or describe all of them herein. The salient and important results and markers related to my MM treatments are already mentioned in Tables 1-3. However, all therapies, including the chemotherapeutic cycles that were used throughout my treatment since the day of diagnosis, are summarized in Table 4.

**Table 4:** Line of therapies for my multiple myeloma treatment in chronological order since the day of its diagnosis CyBorD: cyclophosphamide, bortezomib, dexamethasone; Dex: dexamethasone; DKD: carfilzomib, dexamethasone, daratumumab; KRD: carfilzomib-lenalidomide-dexamethasone; FLC: free light chain

Line of therapy	Date	kappa mg/L (FLC)	kappa/lambda ratio
Diagnosis	December 15, 2019	18,200	319
CyBorD 12 cycles	till February 2, 2020	2,140	75
Bortezomib/Dex 12 cycles	till October 21, 2021	2,270	84
Drug Holiday	till January 22, 2022	8,250	640
DKD x9 cycles	till October 20, 2022	1,940	161
KRD x5 cycles	till April 6, 2023	567	27
Teclistamab since May 2023 and still receiving	December 4, 2023	1.94	1.1
	January 15, 2024	2.69	4.01
	February 25, 2024	3.75	1.78
	March 15, 2024	11.60	9.13

**Figure 2:**
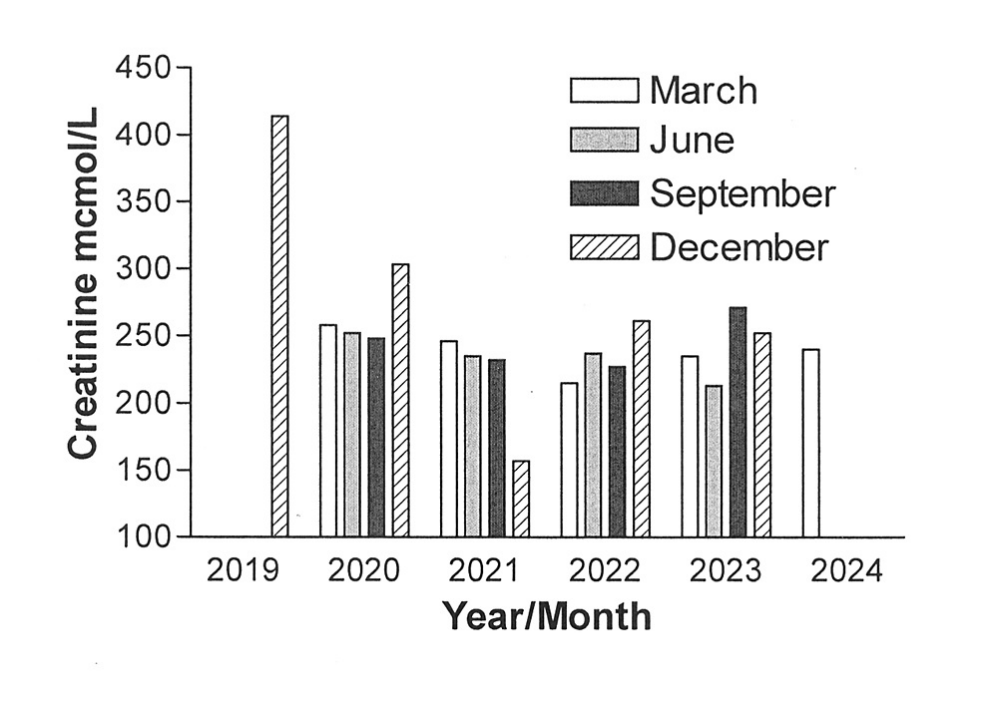
Creatinine level observed from December 2019 to December 2023 during the treatment course of multiple myeloma Note: Please note that December 2019 was the diagnosis date for multiple myeloma.

All results presented in Tables 1-4 have been discussed appropriately in the in the above section. The most important markers for monitoring MM are regular testing of the kappa FLC levels and kappa/lambda ratios. The results for this FLC level of kappa throughout the treatment period are shown in Figure 1A and the kappa/lambda in Figure 1B. Creatinine is another significant marker to measure the nephrological functioning of the kidneys. Figure 2 presents the creatinine level measured throughout my treatments.

## Discussion

Observations on the prognosis of my MM treatment give me great hope for an increased survival rate with different combinations of medications. Researchers worldwide are making efforts to combine certain drugs that have the least side effects and an extended period of progression-free survival (PFS) [12]. The duration of my treatment of nearly four years since the diagnosis of MM shows evidently (Tables 1-4), treatment milestones as discussed above in the treatment section. The present results reflect the disease progression/regression patterns, and the significance of specific laboratory markers by constant monitoring of the most significant marker (kappa FLC and kappa/lambda ratio) throughout this duration. All laboratory tests especially the monitoring of kappa FLC and the kappa/lambda ratio provided evidence for adopting the appropriate treatment cycle protocols as and when it necessitated (Tables 1-4), conforming to the earlier studies [2,10-12]. Furthermore, the observed increased level of kappa FLC and the kappa/lambda ratio after the drug holiday period indicated the relapsing stage of my MM (Figures 1A, 1B, 2). It also formed the reason for selecting teclistamab as one of the possible options for handling this disease. Among many bispecific antibody therapies, teclistamab is one of the most potential medications currently studied in numerous monotherapy and combination therapy trials [16]. Teclistamab stands as the latest addition to a growing battery of immunotherapies that target cellular immunity to treat relapsed MM [16].

The results in Table 4 and Figures 1A, 1B, 2, evidently suggest the effectiveness of teclistamab after relapse of my MM. Although at the initial stage of my treatment with the teclistamab response remained insignificant, later, as a delayed response, it began its effectiveness by dramatically decreasing the kappa FLC level and kappa/lambda ratio level, and until now, the kappa FLC is within a normal range (Figures 1A), and also, the kappa/lambda ratio is maintained at a constant low level (Figure 1B). It is pertinent to mention here that a phase I study (NCT03145181) evaluating the efficacy and safety of teclistamab in patients with R/R MM has shown that teclistamab is well tolerated at a subcutaneous dose of 1.5 mg/kg once a week, with no discontinuations due to treatment-emergent adverse events supporting further clinical development [10]. Additionally, an international open-label phase 2 expansion study of teclistamab in patients with R/RMM is also underway (NCT04557098) that has shown a high rate of deep and durable response in patients with triple-class-exposed R/R MM [17]. Furthermore, it is a reported fact that the burden of infections remains substantial with teclistamab, possibly, due to treatment-induced immunosuppression [14]. The incidence of severe CRS/ICANS was found to be higher in the real world compared to what was seen in clinical trials, emphasizing the use of primary IVIG prophylaxis, which can significantly lower infection-related morbidity and mortality [16]. Receiving monthly IVIG support during my treatment with teclistamab is by having a control on getting infections [17]. In my case, at present, I am receiving 1.5 mg/kg of teclistamab once every two weeks with a monthly administration of IVIG with no observable side effects, which in itself is an encouraging outcome to date. Apart from my personal experiences with teclistamab treatment, it remains important to realize the side of caution as patients' experiences with the response and side effects of teclistamab can vary widely. However, it appears that the continuous advancement in new immunotherapy drugs has instilled fresh hope for achieving a better result for MM [18-20]. At the time of writing this article, the FLC level and the kappa/lambda ratio are within normal range and my treatment is still ongoing.

Among the various other parameters observed during the prognosis and treatment of my MM, creatinine is considered a reliable marker to measure the status of kidney functions. Observations on creatinine level from the day of diagnosis to date clearly show that although the level is at a higher level than the normal range, it should be noted that it remains at a plateau range throughout the treatment (Figure 2). This reflects the minimal side effects of my treatment medications throughout. However, there is no problem experienced in urination.

## Conclusions

The purpose of compiling the salient features and results of my prognosis of MM treatment is to emphasize the visibility of successful treatment with teclistamab. Although MM has not been claimed to be cured, teclistamab represents a possibility of longer follow-ups with maintained disease-free status. Although the effect of teclistamab, especially on R/R MM, has already been studied and established in several other trials and real-world studies, my article might contribute some positivity to justify the reported trials with teclistamab that include measurement of minimal residual disease with identification of the best therapeutic approach for R/R MM soon.
